# Case Report: Design of a University of the District of Columbia (UDC) intrepid dynamic exoskeletal orthosis

**DOI:** 10.3389/fresc.2025.1597923

**Published:** 2025-07-16

**Authors:** Ji Chen, J’niya Butler, Mawussi Nzonou, Lara Thompson

**Affiliations:** Biomedical Engineering Program, Department of Mechanical Engineering, University of the District of Columbia, Washington, DC, United States

**Keywords:** IDEO, UDC workflow, prototype, evaluation, joint angle, spatiotemporal measures

## Abstract

Intrepid Dynamic Exoskeletal Orthosis (IDEO), developed in Walter Reed National Military Medical Center (WRNMMC), is a custom, energy-storing orthosis made of carbon fiber. It is designed to improve mobility of individuals who have ankle dorsi and plantar flexor weakness. The development of IDEO follows a traditional workflow which is time consuming and costly. Users have reported functional limitations due to structural and material rigidity. This case report introduces a new IDEO design workflow which aims to achieve device functionality comparable to the IDEO from WRNMMC, and to reduce cost and time of production. Two IDEO prototypes were created. An experimental evaluation was conducted on a healthy participant wearing prototype 1 to assess the effects of IDEO on task completion time, joint angle and spatiotemporal measures. The participant walked along 3-meter straight line back and forth three times at self-selected speed in two conditions: walking without IDEO (baseline condition) and walking with IDEO. Our results show that the IDEO either hindered the movement or impaired the dynamic balance based on the current outcome measures, except that the range of motion of left ankle dorsi and plantar flexion angle was significantly reduced by 11 degrees (*p* value <0.001). Prototype 2 was created to compare time and cost of device production between UDC and WRNMMC workflows. The analysis shows that our approach reduced the cost and time. Further clinical evaluation is needed to assess the overall functionality of both prototypes when compared to the WRNMMC IDEO.

## 1 Introduction

**Figure 1:**
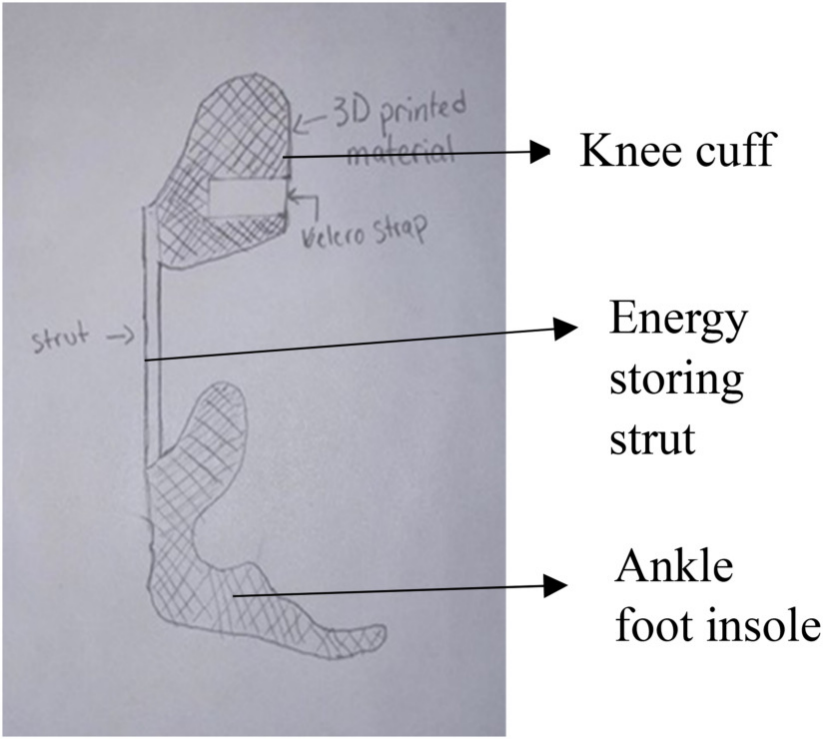
IDEO brace.

Intrepid Dynamic Exoskeletal Orthosis (IDEO) brace developed in the US Army research centers, is a custom, energy-storing carbon fiber orthosis designed to improve gait, stability and function (1). The device includes a patellar tension-bearing knee cuff and a supramalleolar ankle foot orthosis insole, connected by a carbon fiber energy storing strut in between as shown in Figure 1 (1). By redistributing weight and pressure throughout the lower leg and alleviating pain, the IDEO brace is designed to assist the patient in walking, running, and other daily activities of living. A research study on the effects of IDEO use on desire for amputation shows that the IDEO brace improves functional performance related to walking and running and that the majority of subjects initially considering amputation favored limb salvage after using the brace (1). A review study shows that in 213 patients with 222 lower extremities treated with an IDEO brace, at the one-year follow-up, 116 (61.1 percent) of the 185 limbs with use data available reported regular brace use (2). It also shows that 37 limbs (15.7 percent) reported occasional use (2). In addition, this study states that brace efficacy depends on the rehabilitation strategies, pain levels and initial diagnosis (2).

**Figure 2:**
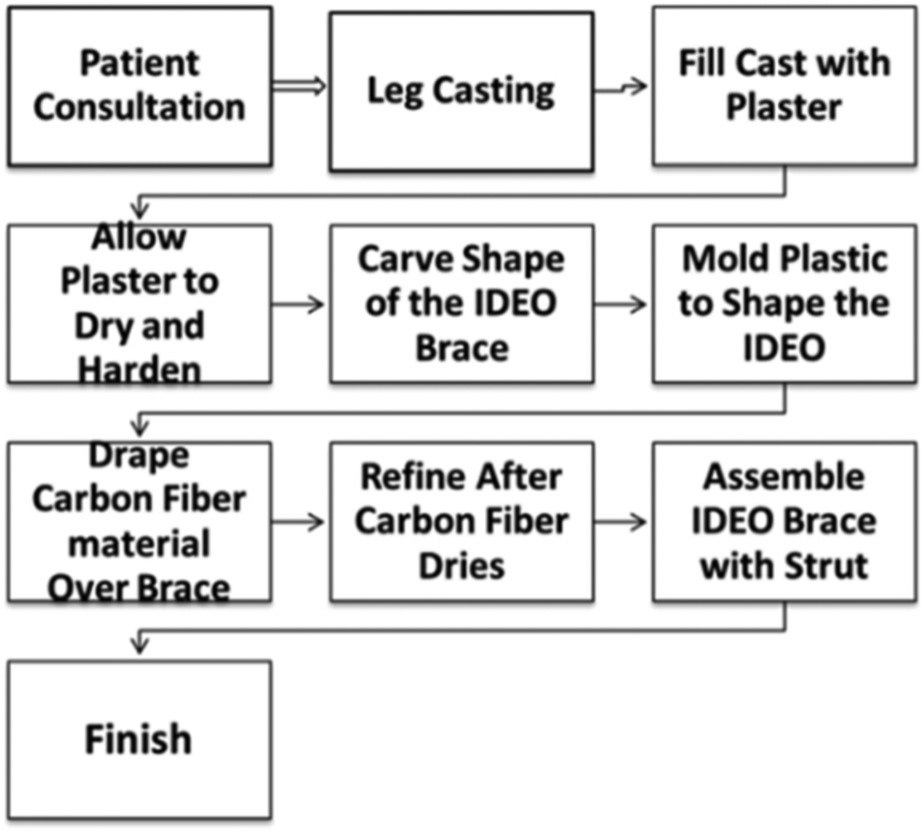
WRNMMC IDEO design workflow.

While the IDEO brace can reduce the desire for amputation and increase the activity level, the current brace design process at Walter Reed National Military Medical Center (WRNMMC) contains a few limitations. Based on its design workflow as shown in Figure 2, for a very experienced prosthetist, it can take over 10 h in terms of initial casting and fitting, likely longer in that sometimes multiple fitting iteration may be necessary. Then laying and curing the carbon fiber would take another 6 h. In addition, the carbon components need to be post-processed and then assembled, leading to additional time. The brace was constructed of lightweight black carbon fiber and was custom fit to patient. A shoe foot plate is connected to a strut that travels up the back of the calf to a knee cuff. A common concern is the thick or bulky feel of the brace while inserted into shoes. As a result, the prosthetist often needs to go through a tedious process to make the brace with minimum bulkiness. In addition, some patients have reported strut restrictions while driving and attempting to accelerate or when braking due to material rigidity.

**Figure 3:**
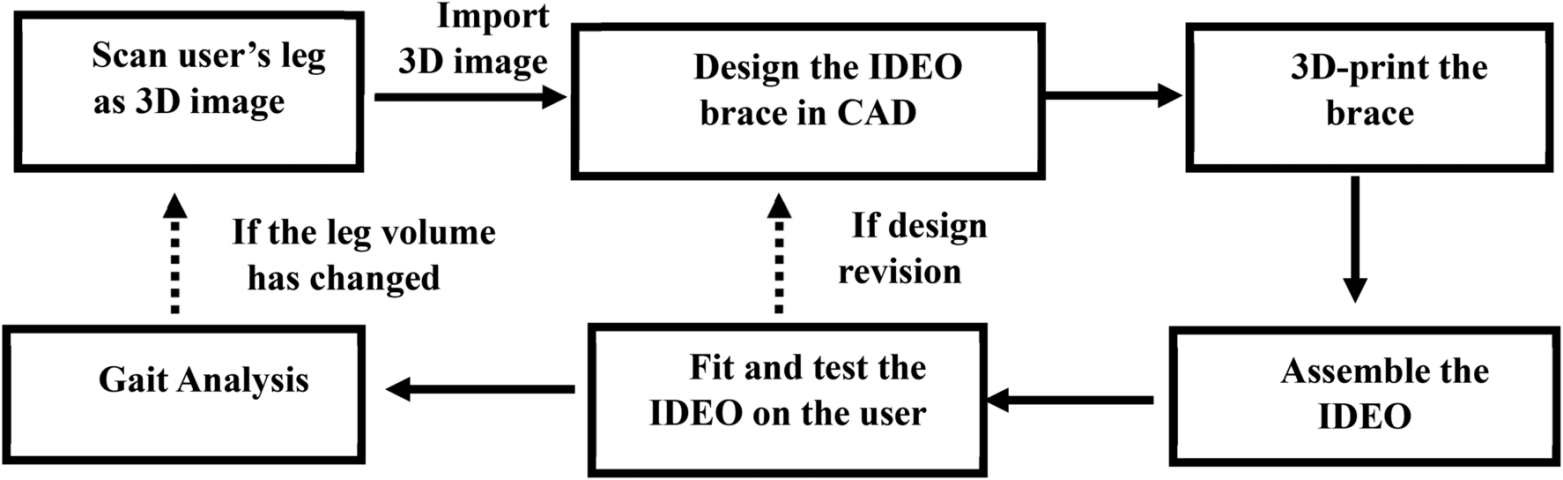
UDC IDEO design workflow.

The objective of our project is to improve the WRNMMC IDEO design process by introducing the UDC IDEO design workflow (Figure 3) that can utilize computer aided design technologies, mechanical modelling and gait analysis. Our two design goals are to create and evaluate a UDC IDEO prototype.

Two prototypes (1 and 2) were fabricated out of the UDC design workflow. We have also created an experimental protocol to evaluate the feasibility of our IDEO prototype 1 on a healthy participant during a walking task. The objective of this evaluation study was to examine the change of mobility and joint angles when walking with IDEO in healthy participants before recruiting the participants who have dorsi or plantar flexor muscle weakness or plantar flexor muscle spasticity for a larger multi visit study. We hypothesized that when comparing to baseline condition (without IDEO brace) for a healthy participant, (1) averaged peak ankle dorsi and plantar flexion angles across gait cycles on both legs would be less; (2) averaged peak knee extension angles across gait cycles will be less (i.e., more extended) during the stance phase; (3) the average gait speed will be greater when walking with the IDEO brace.

## 2 Methodology

Our design process relies on state-of-art technologies, which include the use of a 3D body scanner to scan a human leg, and the use of two CAD software (nTopology and CREO) to conduct the mechanical design of parts and assembly, and then the use of motion capture system to assess the effects of IDEO on gait kinematics and dynamic balance.

### 2.1 Ethics statement

One healthy participant (24 years old, 160 cm, 54 kg) was recruited under University of the District of Columbia (UDC)'s Institutional Review Board approved protocol (2226663-1). An informed consent was obtained prior to data collection. We have obtained informed consent for the publication of deidentified image and data in compliance with CARE guidelines for the case report.

### 2.2 Mechanical design

We 3d-printed the knee cuff and ankle foot insole of our IDEO prototype 1 using Acrylonitrile Butadiene Styrene (ABS), instead of the carbon fiber used by the WRNMMC. Compared to carbon fiber, ABS is less expensive, more easily molded as being thermoplastic and more flexible, which makes it a great candidate for knee cuff and ankle foot insole with respect to user comfort. Currently there is a lack of clinical trial data focusing on the comparison of ABS vs. carbon fiber use in knee cuffs and ankle foot insoles in terms of three features relevant to feasibility: durability, long-term safety and fatigue resistance. Moreover, even for mechanical properties such as ankle foot orthosis (AFO) stiffness, lacking standardization, differences in fixturing and alignment practices can make measured stiffness vary greatly (3). However, a research focusing on ABS mechanical properties has provided evidence to support its use in AFO fabrication, in which the mechanical stiffness and energy dissipation of 3D printed AFO components were found to be like prefabricated carbon-fiber components (4). Another study shows that sufficient elasticity and durability can be provided by printed ABS AFO prototype (5). AFOs often undergo cyclic loading while walking. A significant amount of loading is applied to the strut component. Therefore, the strut of our IDEO brace is still made of carbon fiber to provide structural rigidity and excellent fatigue resistance, and to store and return energy to assist the walking.

**Figure 4:**
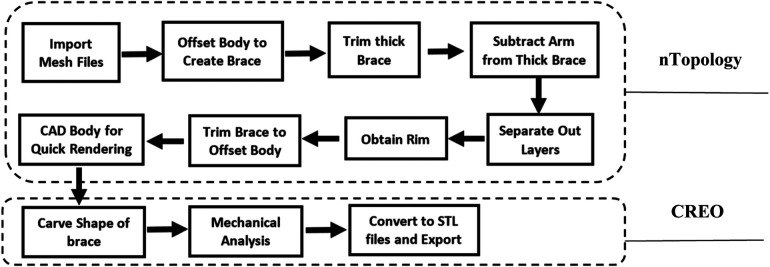
Computer aided design (CAD) modelling pipeline. The top block includes eight steps which are performed using nTopology software. The bottom block includes three steps which are performed using CREO software.

**Figure 5:**
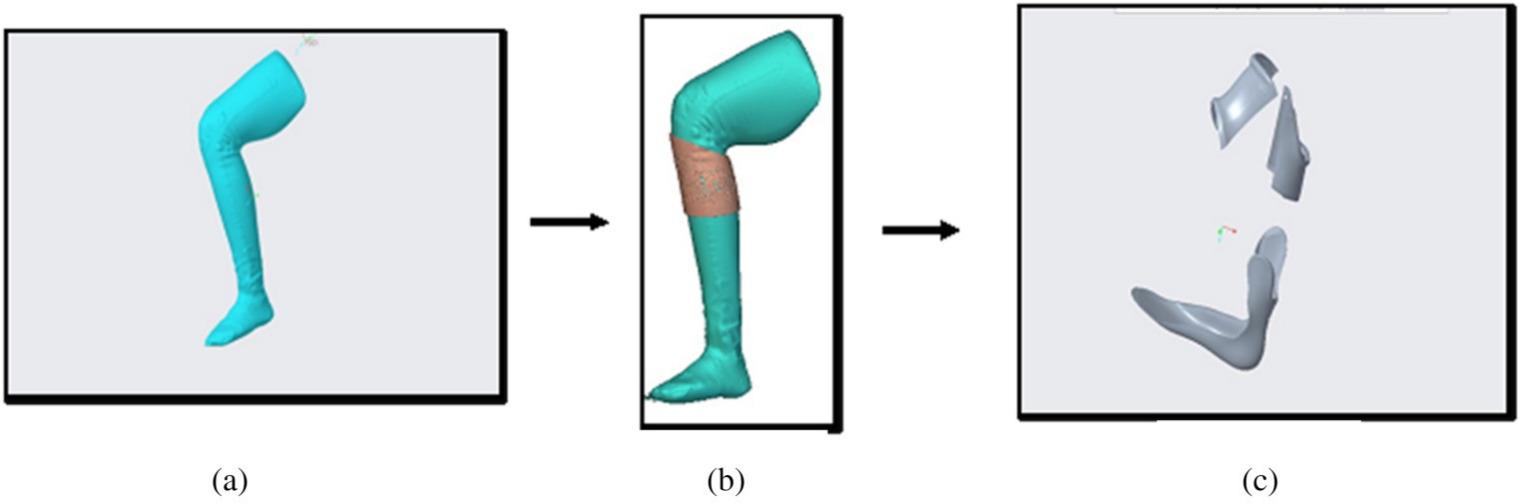
CAD based IDEO design flow: **(a)** scanned 3D model of the participant's thigh, lower leg and foot from left side; **(b)** design of left knee cuff in nTop; **(c)** finalized parts of knee cuff and ankle cuff in CREO.

The CAD based mechanical design pipeline is shown in Figure 4. The steps performed in nTopology are based on the guideline of custom 3D-printed casts from 3D scan data (6) developed in nTopology software (nTop, New York, NY). An exemplary IDEO CAD rendering process is shown in Figure 5. The original IDEO procedure is estimated to take 1–2 weeks to fully design due to the mold casting of the leg and carving the outline of the IDEO by hand. Once the design is finalized the device production can take between 2 and 3 weeks. The entire production pipeline shown in Figure 4 is expected to expedite the process. The digitally scanned 3D model of human leg (Figure 5a) was first imported into nTopology. And then the offset structures were created on the topology of the shank within the selected region of interest as shown in Figure 5b. We call these offset structures pre-made knee cuff and pre-made ankle-foot insole, which were then imported into CREO to be further carved into desired shapes (Figure 5c). These structures were later 3D printed by using the Fortus 450mc Industrial FDM 3D Printer.

**Figure 6:**
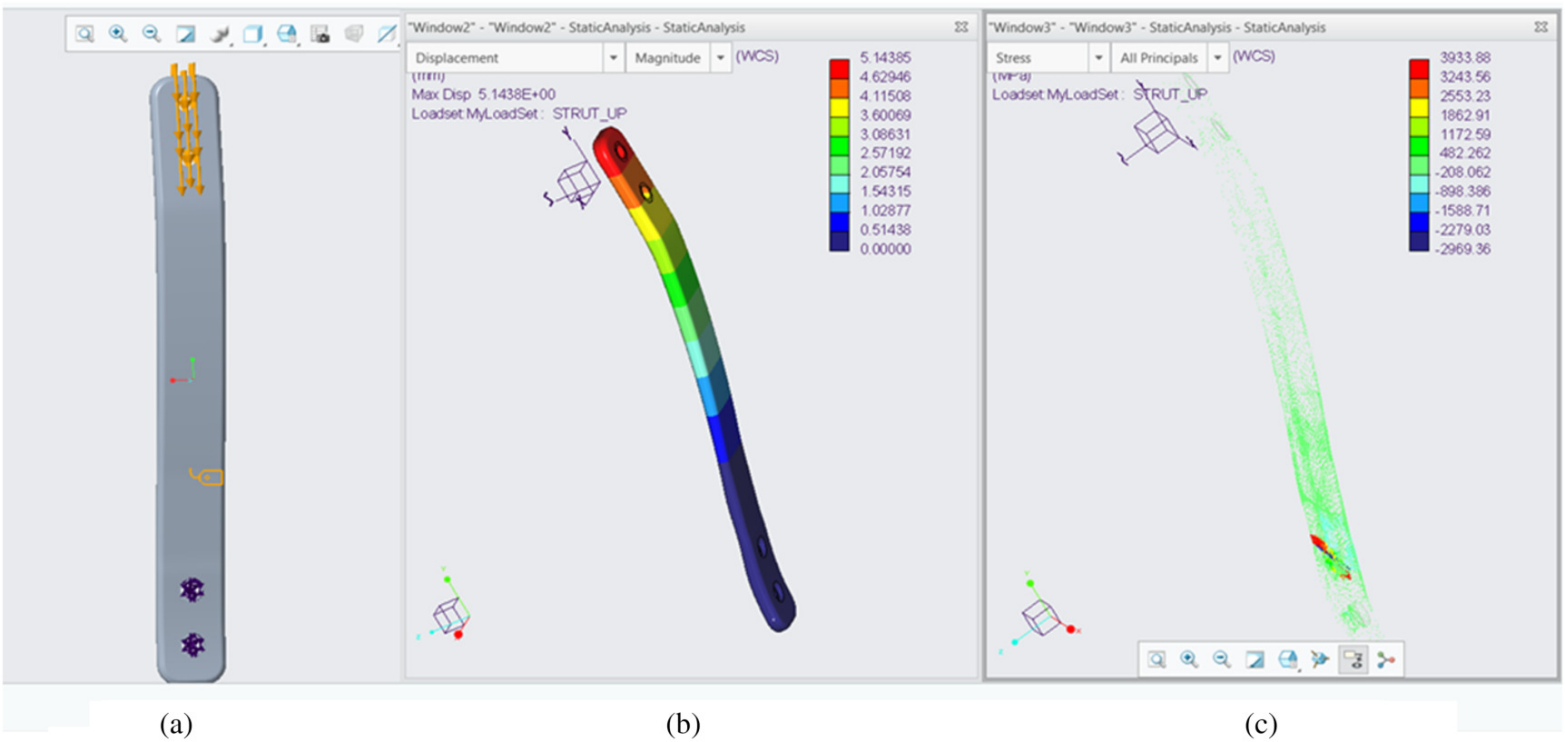
Bucking analysis of the strut used in IDEO prototype. **(a)** Loading configuration; **(b)** the deflection distribution. **(c)** The stress distribution. F = 200 lbs. Material: Carbon fiber G = 250 GPa.

To select the proper strut for IDEO, buckling analysis was conducted in CREO to simulate stress and deflection the IDEO strut experiences under the 200-pound load from a single leg (Figure 6). To determine the load and strut length for simulation, we assumed that the peak amplitude of the vertical ground reaction force (vGRF) in walking can increase up to 1.5 times of body weight (7). As our participant weighs 54 kg, the peak vGRF can then reach to about 180 lbs. We then added some safety margins to set the loading to 200 lbs in our buckling analysis. Moreover, buckling results show that the maximum deflection happens at the end of strut that is connected to the knee cuff, which is about 5 mm. The highest stress distribution is mainly located on the part of the strut that is connected to the foot plate. By using the deflection and stress distribution data, along with the leg length of participant, we selected the 250 mm individual PDE spring (Fabtech, Everett WA) as our brace strut.

### 2.3 Evaluation protocol

**Figure 7:**
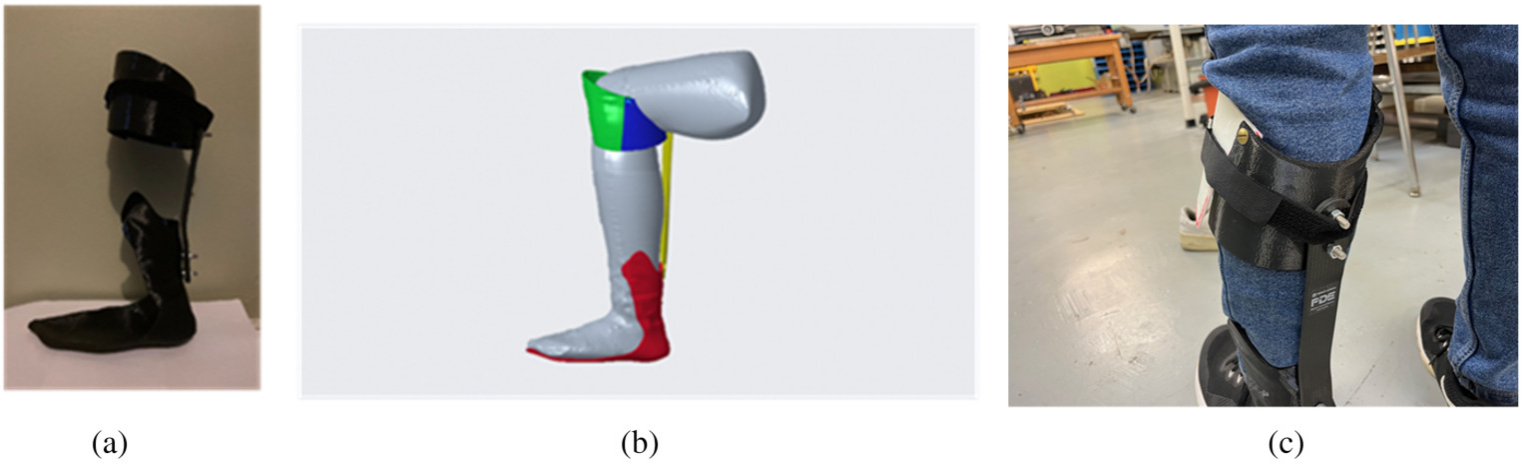
UDC IDEO prototype 1. **(a)** Actual assembly. **(b)** CREO assembly sideview. **(c)** Participant wearing the UDC IDEO.

A protocol was created to evaluate the effect of UDC IDEO prototype I (Figure 7) on gait mechanics during its immediate use. Protype 1 was developed out of the left leg model of the participant. To be eligible for this evaluation study, the participant must be over the age of 18, be able to provide verbal or written consent, and be able to understand and perform the tasks described in the protocol. The participant should not have any neurological or cardiorespiratory condition or diagnosis. Lastly, they should be able to fit into the IDEO brace.

The evaluation consisted of a two-hour visit to the Center of Biomechanics and Rehabilitation Engineering (CBRE) at UDC. The participant was assessed in two conditions: (1) walking without IDEO (baseline condition), and (2) walking with IDEO brace, each of which included two trials. Normally the standardized 10-meter walking test (10MWT) and 6-minute walking test (6MWT) are used to investigate the effectiveness of AFO like IDEO (8). These two tests were not used in our study due to limited space. In addition, it was reported that these two tests do not detect well the effects of ankle foot orthosis on patients with ankle dorsiflexor paresis (9). A short distance (3 meters long) was therefore used in our walking test. It was reported that AFO use may impair the compensatory stepping response for dynamic balance to avoid fall, when events such as making turns and transient state of walking occur (10). A shorter distance with turning may therefore increase the likelihood of detecting change in selected outcome measures. Given that only a few mid steps can be extracted to represent the normal walking conditions for one bout in a 3-meter distance, as an alternative, we asked the participant to walk back and forth along this distance three times in each trial, which is equivalent to 18 meters in total distance. The total six repetitions for each condition also align with the total number of repetitions of straight line walking task reported in other AFO assessment studies (11, 12).

The gait cycle was based on identifying two types of gait events for one foot: heel strike (the start of weight loading) and toe off (the end of weight unloading). Given that the force plates were inaccessible, we closely observed the targeted foot movement in VICON Nexus software to manually label these two events using heel marker and toe marker of the left leg. We labelled the heel strike event when the heel strike marker reached the adjusted ground level in the Nexus environment. The toe off events were labelled when the toe marker started moving away from the adjusted ground level. The steps extracted from each trial include all steps which have two consecutive heel strike events and one toe-off event in between during the walking part. We excluded all steps when the participant was turning around to walk back. The above-mentioned criteria as our routine procedure was used to extract and count steps for all trials. Table A1 in Supplementary Appendix A provides detailed information about extracted gait cycles of all trials.

After walking, the custom brace was removed and the skin area covered by IDEO was inspected for any pressure area from wearing the brace. Feedback was requested from the participant as well.

The study protocol implemented an experimental setup utilizing the VICON motion capture system (Vicon Motion Systems, Oxford, UK) with the sample rate of 100 Hz. Thirty-nine 14 mm markers were placed at bony anatomical landmarks of the full body in accordance with the Vicon Full Body Plug-In-Gait body model (13) for motion capture. Two markers designated for two anatomical landmarks: the ankle joint axis at the lateral side and the left heel, were placed on the relevant locations on the IDEO, as these anatomical landmarks were covered by the IDEO brace during the assistive walking. Our data analysis covered three components: data pre-processing, data storage and data post processing, for which detailed description can be found in (14).

## 3 Results

**Figure 8:**
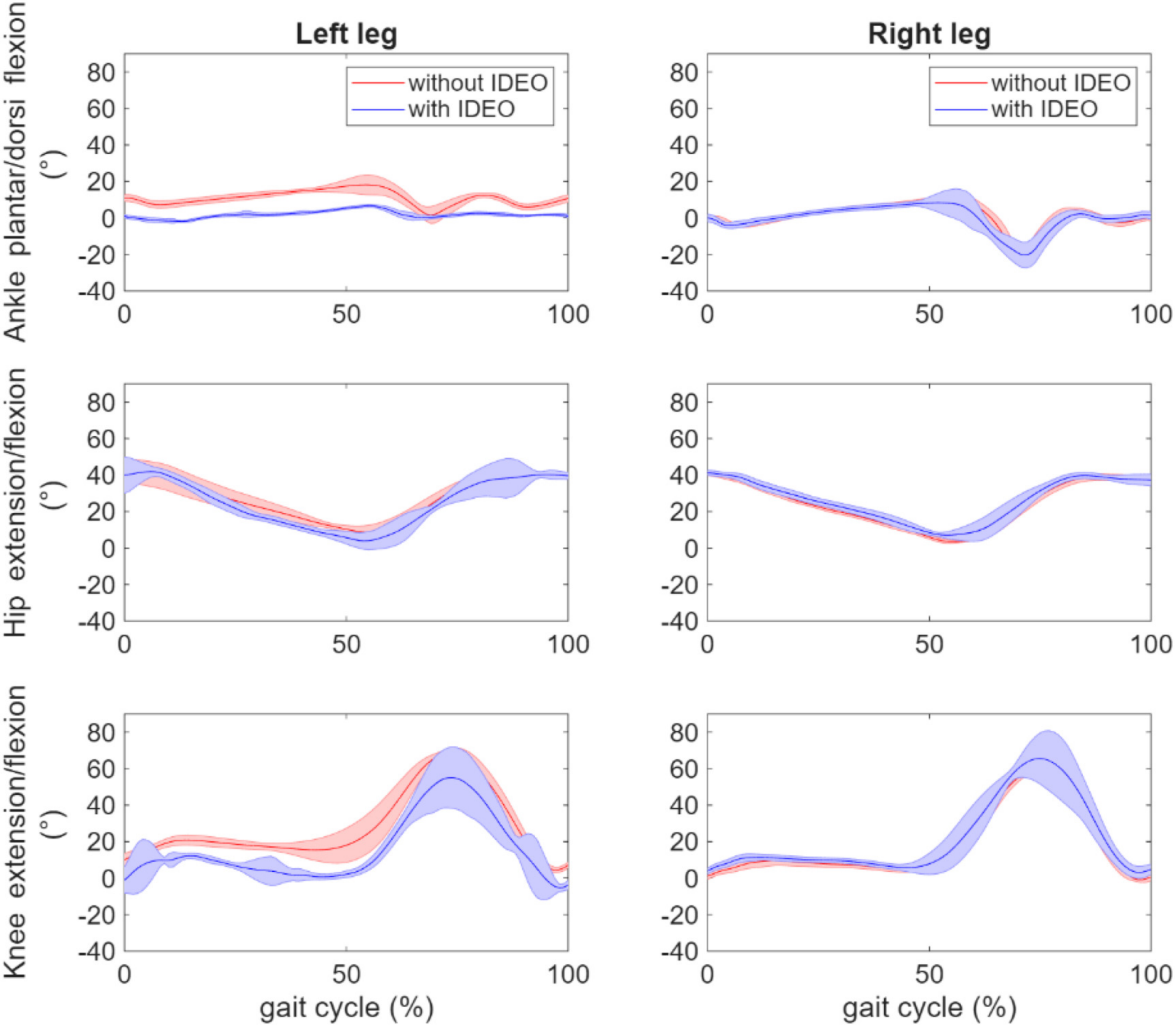
Comparison of averaged hip, knee and ankle joint angle profile across all corresponding gait cycles between two walking conditions: without IDEO (baseline) and with IDEO.

**Figure 9:**
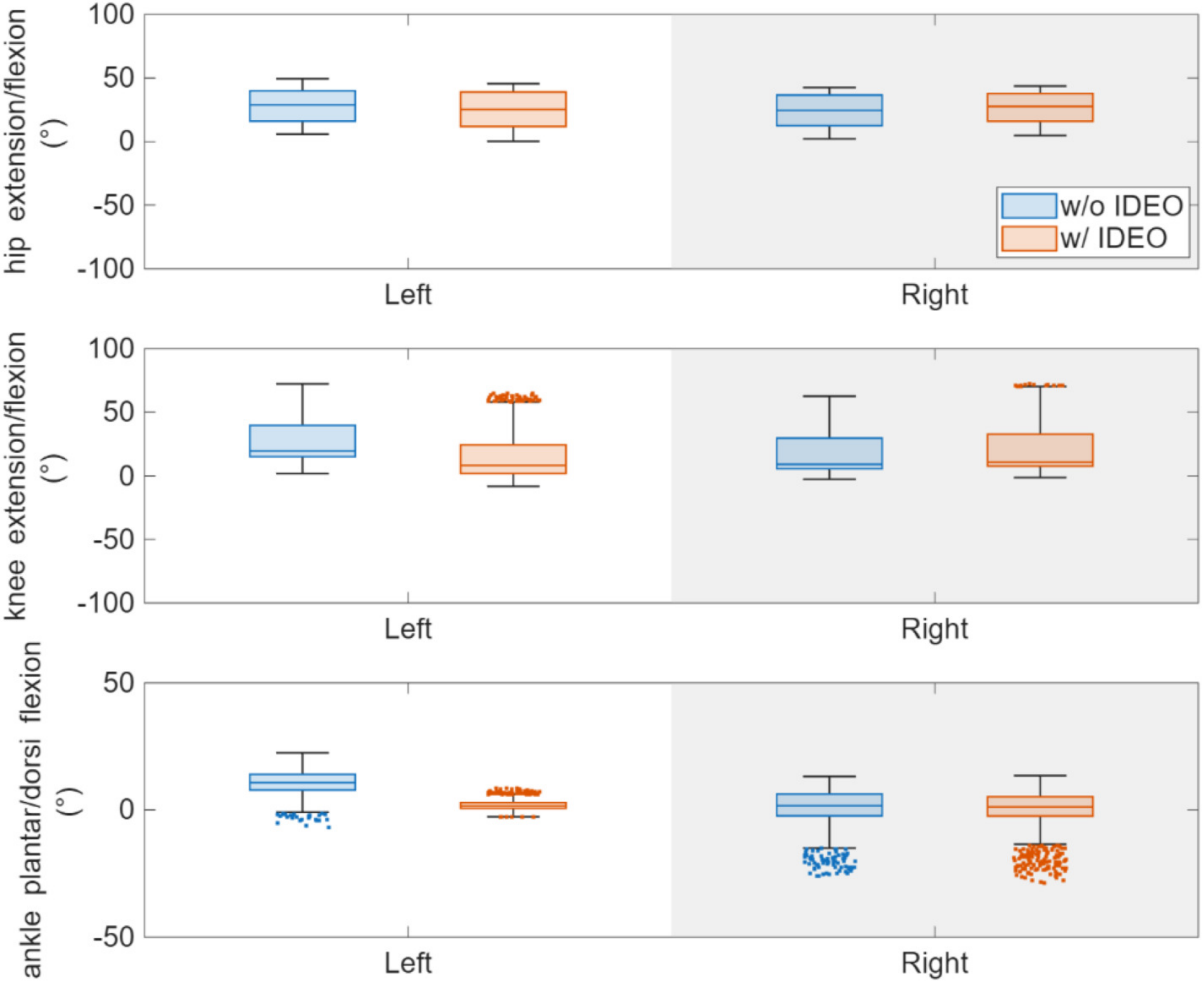
Comparison of hip, knee and ankle angle statistics between two walking conditions. The outliers are jittered with changed marker style for better display. Here positive ankle angle is dorsiflexion, and the negative ankle angle is plantarflexion.

The average task completion time for walking without IDEO and walking with IDEO are 32.5 s and 33.2 s. To compare the joint angle between conditions, we calculated its mean and standard deviation for each condition in the following way. We normalized the time it takes to complete each trial of task to 101 timeframes between 0 and 100. 0 means the start of the task whereas 100 means the completion of task. Linear interpolation was used to normalize the joint angle data with the same size as the normalized time data. These normalized angle data were then averaged across all identified gait cycles for each leg to produce means and standard deviations of joint angles. MATLAB (MathWorks, Natick, MA) data processing scripts were created to generate average joint angle profiles in a gait cycle as shown in Figure 8. We compared two conditions and used MATLAB Boxchart to display the statistics data of all joint angles with the median, the lower and upper quartiles, any outliers and the minimum and maximum values that are not outliers (Figure 9). In addition, a descriptive statistics was conducted to produce minimum (min), maximum (max) and ROM of joint angles shown in Table A2 of Supplementary Appendix A. As mentioned previously, we first produced normalized joint angles from all identified gait cycles. Then we calculated the min, max and ROM of these joint angles for each gait cycle in each condition. Last, we grouped and averaged the min, max and ROM of these joint angles for the side of leg to calculate their means and standard deviations in each condition.

To investigate the effects of IDEO brace on the joint angles, we first took the set of joint angles based on the joint and leg side through the Kolmogorov–Smirnov test to test the null hypothesis on whether or not each set of data comes from a normal distribution. As the null hypothesis was rejected, a ranksum in MATLAB toolbox (equivalent to a Mann–Whitney *U*-test) was performed to compare the joint angles of the left side between two conditions. ROM of left ankle flexion/extension angle significantly decreased by 11 degrees (*p* value <0.001) when walking with IDEO. The left part of Figures 8 demonstrates that ankle flexion and extension was constrained on the left side when walking with IDEO. No significant difference was found in the ROM of the right ankle dorsi and plantar flexion angle, and in ROM of knee and hip flexion/extension angles for both sides between two conditions.

To further investigate the effects of IDEO on walking dynamics, we calculated the averaged values across extracted gait cycles of following measures: cadence, walking speed, step time, and left foot off percentage with respect to gait cycle as shown in Table 1. A ranksum test was performed to compare all these measures between walking with and without IDEO for each leg. No statistical significance was found in any of these measures for each leg.

**Table 1:** Comparison of spatiotemporal parameters.

Testing condition	Walking without IDEO				Walking with IDEO			
Trial No.	1		2		1		2	
Leg side	Left	Right	Left	Right	Left	Right	Left	Right
Cadence (steps/min)	88.9	92.3	93	97.6	88.9	86.3	87.6	87
Walking Speed (m/s)	0.73	0.76	0.8	0.81	0.77	0.73	0.71	0.72
Step time (s)	0.67	0.63	0.63	0.6	0.69	0.66	0.7	0.67
Left foot off (%)	67%	67%	66%	67%	60%	65%	61%	65%
Step length (m)	0.51	0.49	0.53	0.47	0.51	0.51	0.48	0.5

**Figure 10:**
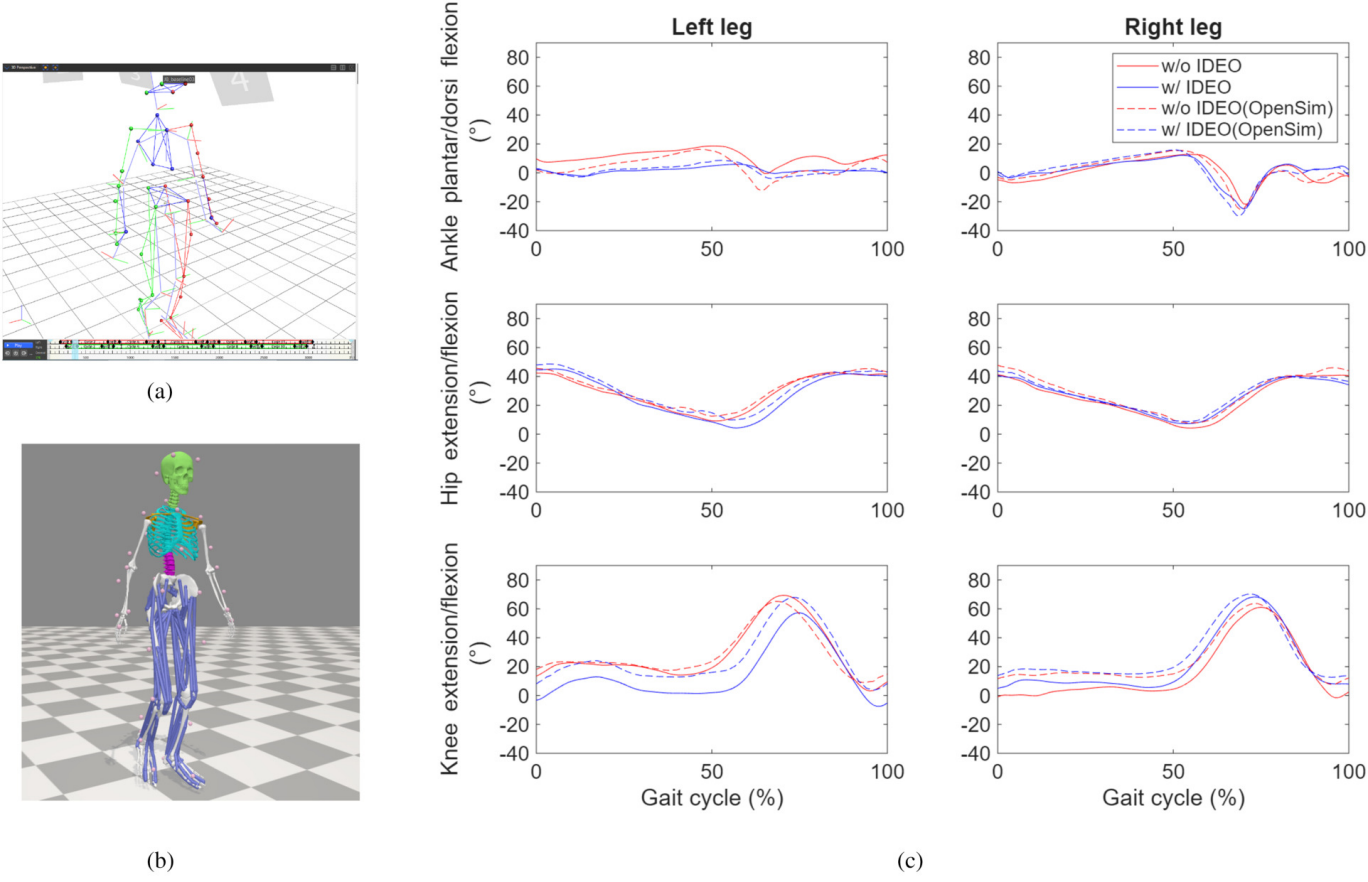
Joint angle profile comparison between VICON pipeline and OpenSim pipeline. **(a)** The subject skeletal model reconstructed in Nexus. **(b)** The subject musculoskeletal model reconstructed in OpenSim. **(c)** Comparison of joint angles processed by both Nexus and VICON for selected time frame from one trial of each condition.

For this study, we have created preprocessing pipelines for markers data of static (calibration) trials and dynamic (walking) trials in Nexus. Data structure was created to store the marker data, angle and anthropometric information of participant. To further expand our analysis ability, a user-specific musculoskeletal model was created using an OpenSim pipeline (15) to conduct and compare its joint angles of one trial from each condition to joint angles calculated in VICON Nexus pipeline shown in Figure 10. As an open-source software, OpenSim can also assess joint reaction forces and moments, and muscle activation with the collected ground reaction forces.

The validity of the OpenSim model was quantified by checking the similarity of its joint angle profiles with respect to the angels generated by VICON Nexus pipeline. Normalized cross-correlation (NCC) coefficient was calculated to assess its temporal similarity between angle profiles. The temporal similarity is considered excellent if NCC coefficient value is great than 0.9, strong if the value is between 0.7 and 0.9, moderate if the value is between 0.4 and 0.7 (16). The NCC coefficients for all but the angles of left ankle in the condition of walking with IDEO are greater than 0.9, which means the excellent similarity of these angle profiles. The NCC coefficient for the angle of left ankle with IDEO is 0.84, which means the strong similarity of angle profiles.

## 4 Discussion

Our analysis shows that the ROM of ankle dorsi and plantar flexion decreased significantly at the left leg, the average peak hip and knee extension angles decreased significantly at the right leg, and the averaged peak knee flexion angles increased significantly for both stance phase and swing phase at the right leg, when walking with IDEO. Some of our hypotheses were rejected. For example, we didn't see the significant reduction in averaged peak ankle dorsi and plantar flexion angles across gait cycles on right leg and in averaged peak knee extension angle from both legs. The average gait speed and other spatiotemporal parameters didn't change significantly. The observed change of joint angle in hip and knee joints may be because the restricted left ankle movement from brace induced compensatory mechanisms in these two joints.

These joint compensations may explain why spatiotemporal parameters were unaffected. Restricted ankle ROM during push-off can be compensated by the increased hip extensor work to propel the body's center of mass and knee extension through the stance phase, and by the increased hip flexor work to initiate the push-off to swing phase (17). Furthermore, the increased knee flexor work during the loading phase of the contralateral side of leg can be compensated by increased knee extensor work of the leg wearing IDEO brace (18). In addition, maintaining her spatiotemporal measures may be also due to the short walking distance and the participant's excellent fitness level.

11° reduction in left ankle ROM may temporarily provide the user who has ankle weakness with increased stability and proprioception which is needed for walking and running. However, in terms of restoring long-term mobility, this level reduction, especially with restricted dorsi flexion, would impair user dynamic balance during locomotion. A couple of WRNMMC IDEO users anecdotally reported lower back pain developed from its regular use, which is consistent with researching finds that the reduced shock absorption at the heel strike of foot is associated with lower back pain (19). As a result, utilizing these compensatory mechanisms may present the patient user with potential harm such as lower back pain in the long run. In future, we would like to design an ankle component with adjusted stiffness in IDEO brace to accommodate the user's increased mobility level (20).

We didn't use the force plate to measure ground reaction force as it was undergoing a repairing process. As a part of ongoing research, the Tekscan Strideway system (Tekscan, Norwood, MA, USA) will be used to measure the vertical ground reaction forces (vGRFs) and center of pressure (COP) trajectories. These measurements can quantify walking symmetry which reflects the level of dynamic balance during walking. In addition, these measurements will be used in the inverse dynamics component (21) of OpenSim pipeline to calculate the joint reaction forces and moments, which can then be used to estimate the assistance torque provided by IDEO through mathematical modelling (22).

In general, there is a paucity of research focusing on using surface electromyography (EMG) to assess muscle co-contraction when walking with IDEO. We didn't conduct these assessments in the current study as our EMG wireless sensors (Delsys, Natick, MA, USA) were undergoing a replacement process. As part of feasibility evaluation, however, the recording of targeted muscle co-contraction patterns during the IDEO use, when comparing to that recording during no brace use, can provide critical evidence on whether an appropriate level of muscle co-contraction has been employed by the user. Studies have shown that AFOs can alter muscle activation and co-contraction patterns in paretic and non-paretic ankle muscles (23–25). It was reported that the use of AFOs decreased the co-contraction index of paretic tibialis anterior (TA) and gastrocnemius muscles (MG) during the swing phase of walking in stroke patients (25). Moreover, reduced co-contraction can lead to improved ankle control and metabolic cost during walking (25). In addition, co-contraction of ankle muscles can be reduced as the postural stability increases during single leg standing (26). In future study, we will use Trigno Avanti sensors to examine the effect of IDEO brace on the con-contraction of TA and MG when the participant performs 10MWT, 6MWT, TUG, FSST and T-Test. We will recruit individuals with at least one of three ankle muscle impairments (plantar flexor weakness, dorsi flexor weakness and dorsi flexor spasticity). These three types often lead to foot drop, which exists as a major symptom among individuals with chronic stroke (17), and those with ankle fractures or vascular injuries in military settings (27).

**Figure 11:**
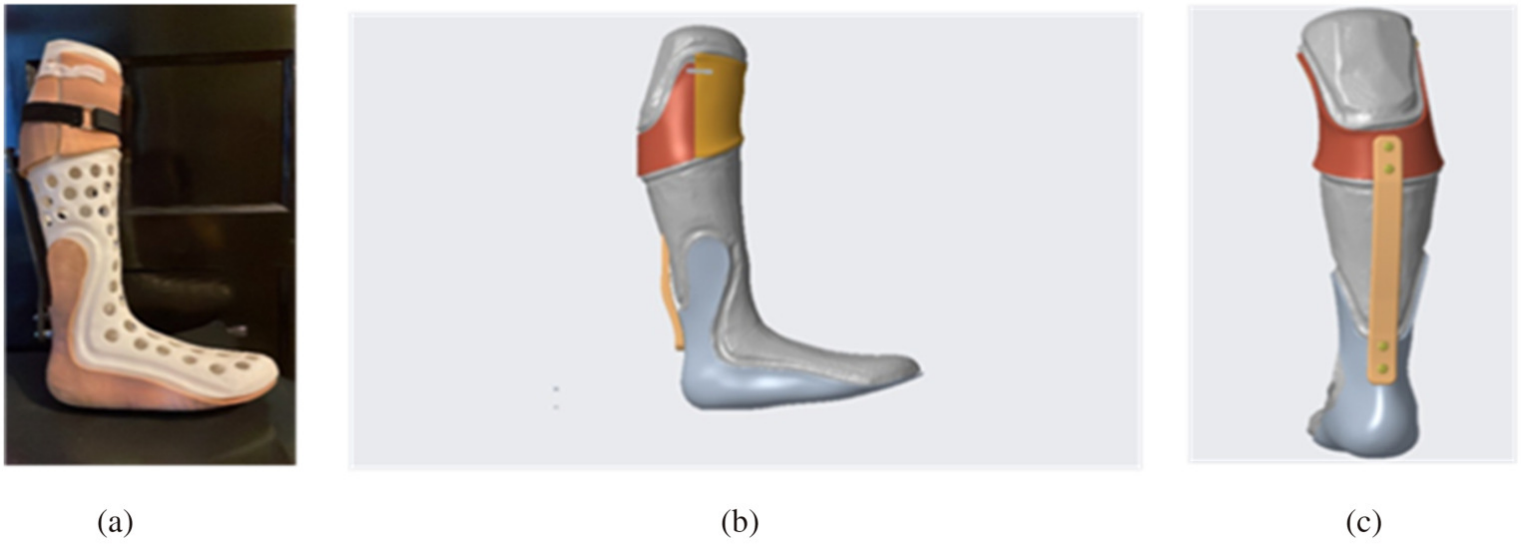
UDC IDEO prototype 2 display. **(a)** assembly display with the actual patient lower leg mold. **(b)** CREO assembly sideview. **(c)** CREO assembly back view.

We also produced the UDC IDEO prototype 2 (Figure 11). It was used to compare with a WRNMMC IDEO brace for cost analysis, as both braces were built upon the same leg model. Compared to WRNMMC counterpart, our UDC IDEO brace weighs about 160 g less, and the estimated cost is about 10% of the commercial price of WRNMMC IDEO, the production time was shortened from about 3 weeks to 3 days. Further evaluation will be needed to provide comparative data on functional performance in terms of biomechanical effects, durability, user comfort, long-term safety and fatigue resistance of brace.

There are two challenges in the current UDC workflow. First the current nTop pipeline doesn't allow us to break the knee cuff to two pieces which are needed for wearing the brace. The knee cuff was then required to be broken into subcomponents in CREO as shown in Figure 5. In the future, we would like to improve the nTop pipeline that can perform functions such as component break-down and connector add-on by following nTop personalized medical devices guide (28). The second challenge lies in the fact that the parts created in nTop need to be converted into a mesh format to be used in CREO, instead of a solid, modifiable geometry. Therefore, digital carving process, which modifies the knee cuff and ankle foot insole components, not only became time consuming, but also didn't produce the very compliant component. In the future, we would like to work with the nTop support team to create a pipeline that can export our mesh as a solid geometry with boundary representation in formats such as STEP or Parasolid.

We will employ the user centered design into the IDEO prototype to facilitate its adaptation in impaired populations such as stroke patients and amputees. In the user centered design, we will modify individual components of IDEO brace to meet evolving demands of user mobility and comfort, based on the user feedback and performance progression. In addition, we will also facilitate its adaptation by developing task specific practices.

Currently the participant was only asked to perform forward walking and turning along a straight line. More challenging tasks should be included to assess dynamic balance and mobility. Furthermore, sufficient practice trials associated with the IDEO brace, along with a subsequent user feedback procedure, were lacking in the current project to adequately capture the user's satisfaction with the device. To address the lack of user feedback, we would like to conduct practice trials in a two-week period to collect feedback from user, a certified prosthetist/orthotist (CPO) and physical therapists about device and therapy approaches. Two-week practice duration has been used to assess the overall device performance before its prescription (29–31). We will work with a CPO at Medical Center Orthotics & Prosthetics (MCOP) (Silver Spring, MD, USA) and therapist at Medstar Health Orthopedics and Sports Medicine (Washington, DC, USA) to develop task specific practices. MOCP has worked with some of us on developing an evaluation protocol for a commercial prosthesis (32). MedStar Health has worked with some of us on developing practice tasks for orthosis and exoskeleton (33–35).

Evaluation of outcome measures will be performed in two timepoints which are before and after a two-week practice. At each timepoint, we will collect data on user-reported outcomes using Orthotics Prosthetics User surveys (OPUS) (36). Users will complete three OPUS modules: Lower Extremity Functional Status Management (LEFS) and Client Satisfaction with Devices (CSD), Client Satisfaction with Services (CSS). With improved testing environment, we will also collect data in performance-based measures such as 10MWT, 6MWT, Timed Up and Go (TUG), a four-square step test (FSST) and T-Test. 10 MWT and 6MWT are designed to test the user's walking speed and endurance. We will also collect gait mechanics in these two tests. TUG test with the IDEO can monitor the change of mobility and dynamic balance over time based on the task completion time (37). FSST is designed to test dynamic balance during rapid stepping forwards, backwards, and sideways over small objects. T-Test is designed to test dynamic balance in forward and backwards runs as well as side shuffles in our evaluation protocol (11, 38).

We performed descriptive statistics to find mean and standard deviation of joint angles in normalized gait cycle which are shown in Figures 8, 9. Mean and standard deviations of peak joint angle and range of motion of these joint angles were also calculated and tabulated in Supplementary Table A2. We found that the left ankle ROM was reduced by 11 degrees, which matches with the participant's note about restricted left ankle movement when walking with IDEO wore on her left leg. To investigate the effect of IDEO, we also conducted a ranksum test to compare the peak joint angle and spatiotemporal measures between conditions for each leg. Although results from this test didn't show significant change, with only 1 healthy participant, we understand it is not statistically appropriate to generalize findings from our statistical inference to all healthy participants.

To ensure adequate statistical power in future studies involving impaired participants, we will improve our study in the following aspects. First, we will increase the sample size. A power analysis will be used to determine the appropriate sample size for future studies based on anticipated effect sizes and desired power levels. Second, we will optimize effect size by using these performance based tasks such as 10MWT, 6MWT, TUG, FSST and T-Test. The goal for this optimization is to increase the probability of detecting the immediate effect of wearing IDEO braces on gait patterns. Third, we will evaluate the effect of IDEO brace on gait patterns after the above mentioned two-week practice. Fourth, the impaired participants may be assigned into subgroups based on the type of ankle impairment to further improve power. Finally, we will improve the robustness of our evaluation protocol to ensure consistent and accurate data collection.

OpenSim tool was also used in this study to assess joint angle profiles of human gait. NCC coefficient of 0.84 with IDEO brace on the left leg falls short of the excellent classification thresholder of 0.9. The reason for this discrepancy is that the left ankle marker placement in the condition of walking with IDEO is slightly different from that marker placement in the baseline condition as the anatomical landmark for the ankle joint was covered by the left ankle foot insole of the brace in IDEO condition. On the meanwhile, the virtual marker placement in the OpenSim model was kept the same for both conditions. Therefore, the NCC score for the left ankle drops below 0.9 in the IDEO condition. Currently we are using the joint angles calculated from VICON Nexus based data processing pipeline (not from OpenSim based pipeline) to conduct comparison analysis between two conditions with respect to our hypotheses. Therefore, the NCC scores don't have impact on our conclusions. However, we plan to use the OpenSim based pipeline to estimate joint load and targeted muscle activation once we measure the ground reaction force and muscle activation in the future study. To ensure the accurate estimation, we would like to address this discrepancy by modifying the ankle foot insole design to make the anatomical landmark of ankle joint in the lateral side completely exposed for marker attachment.

In summary, future researchers should consider a few key limitations identified in this research. First, the data of vGRFs and COP trajectories, and the data of muscle activations, was not collected to quantify the dynamic balance and muscle co-contraction patterns respectively. Second, the participant with ankle muscle impairment needs to be recruited to evaluate the feasibility of the IDEO brace for targeted populations. Third, future research will need to include more challenging tasks such as FSST, TUG and T-Test and may need to increase the number of trials to improve the confidence of statistical assessment of changes as both healthy and impaired participants will be recruited. Lastly, metrics of user satisfaction such as OPUS should be established to quantify the user experience.

## 5 Conclusion

We have introduced a new IDEO design workflow which uses digital scanning, computer-aided design and 3D printing which aims to reduce the cost and time of IDEO development. Two IDEO prototypes (1 and 2) were fabricated. An experimental evaluation was conducted on prototype 1 to assess the effects of IDEO on task completion time, joint angle and spatiotemporal measures of a healthy participant. The ROM of left ankle flexion/extension angle was significantly reduced by 11 degrees (*p* value <0.001) when walking with IDEO. This ankle reduction may have induced observed joint angle differences in knee and hip via compensatory mechanisms. More outcome measures such as vGRFs, EMG, joint force and moment produced by the modelling technique such as Opensim, along with more challenging tasks, will be used to assess the effects of IDEO brace on dynamic balance and mobility as we are recruiting impaired participants. Prototype 2 was used for direct comparison of time and cost of device production between UDC and WRNMMC workflows. The analysis shows that our workflow significantly reduced the cost and time. However, there is certainly more work needed to validate the overall feasibility of both our prototypes when comparing to the WRNMMC IDEO.

## Data Availability

The original contributions presented in the study are included in the article/Supplementary Material, further inquiries can be directed to the corresponding author.
