# Supplementary material for: Case Report: Design of a University of the District of Columbia (UDC) intrepid dynamic exoskeletal orthosis
Source: Front Rehabil Sci. 2025 Jul 16;6:1597923. doi: 10.3389/fresc.2025.1597923 (PMC12307379; doi:10.3389/fresc.2025.1597923)
Supplement: Supplementary file 1 [file Table1.docx]

# Appendix A

Table A1: Detailed information about extracted gait cycles for each leg of all trials

| Trial No. | Walking w/o IDEO trial 1 | | | | | | Walking w/o IDEO trial 2 | | | | | | Walking with IDEO trial 1 | | | | | | | Walking with IDEO trial 2 | | | | | |
| --- | --- | --- | --- | --- | --- | --- | --- | --- | --- | --- | --- | --- | --- | --- | --- | --- | --- | --- | --- | --- | --- | --- | --- | --- | --- |
| Gait Cyle  No. | Left leg  (time frames) | | | Right leg  (time frames) | | | Left leg  (time frames) | | | Right leg  (time frames) | | | Left leg  (time frames) | | | | Right leg  (time frames) | | | Left leg  (time frames) | | | Right leg  (time frames) | | |
|  | heel strike | toe off | next heel strike | heel strike | toe off | next heel strike | heel strike | toe off | next heel strike | heel strike | toe off | next heel strike | heel strike | toe off | next heel strike | | heel strike | toe off | next heel strike | heel strike | toe off | next heel strike | heel strike | toe off | next heel strike |
| 1 | 166 | 256 | 301 | 234 | 321 | 364 | 214 | 299 | 343 | 280 | 362 | 403 | 249 | 330 | | 384 | 176 | 266 | 315 | 304 | 387 | 441 | 233 | 323 | 371 |
| 2 | 301 | 384 | 427 | 768 | 854 | 897 | 749 | 826 | 879 | 679 | 760 | 811 | 801 | 881 | | 932 | 735 | 818 | 871 | 743 | 832 | 884 | 819 | 898 | 955 |
| 3 | 699 | 786 | 832 | 1319 | 1408 | 1454 | 1181 | 1265 | 1314 | 1251 | 1328 | 1375 | 1362 | 1440 | | 1492 | 871 | 953 | 1001 | 1285 | 1368 | 1421 | 1354 | 1434 | 1482 |
| 4 | 832 | 920 | 967 | 1897 | 1978 | 2024 | 1733 | 1817 | 1867 | 1801 | 1880 | 1934 | 1901 | 1983 | | 2032 | 1293 | 1377 | 1428 | 1838 | 1919 | 1975 | 1907 | 1988 | 2039 |
| 5 | 1253 | 1344 | 1387 | 2437 | 2520 | 2566 | 1867 | 1943 | 1997 | 2356 | 2432 | 2479 | 2032 | 2117 | | 2168 | 1428 | 1516 | 1561 | 2379 | 2460 | 2513 | 2449 | 2528 | 2574 |
| 6 | 1397 | 1475 | 1524 | 3005 | 3086 | 3140 | 2291 | 2368 | 2417 | 2845 | 2925 | 2982 | 2678 | 2731 | | 3162 | 1969 | 2048 | 2128 | 2933 | 3016 | 3068 | 3002 | 3078 | 3134 |
| 7 | 1831 | 1912 | 1959 |  |  |  | 2910 | 2994 | 3047 |  |  |  | 3162 | 3238 | | 3293 | 2524 | 2610 | 2670 | 3068 | 3146 | 3198 |  |  |  |
| 8 | 1959 | 2047 | 2092 |  |  |  |  |  |  |  |  |  |  |  | |  | 3088 | 3176 | 3227 |  |  |  |  |  |  |
| 9 | 2370 | 2459 | 2504 |  |  |  |  |  |  |  |  |  |  |  | |  |  |  |  |  |  |  |  |  |  |
| 10 | 2504 | 2588 | 2635 |  |  |  |  |  |  |  |  |  |  |  | |  |  |  |  |  |  |  |  |  |  |
| 11 | 2936 | 3026 | 3073 |  |  |  |  |  |  |  |  |  |  |  | |  |  |  |  |  |  |  |  |  |  |
| 12 | 3073 | 3155 | 3209 |  |  |  |  |  |  |  |  |  |  |  | |  |  |  |  |  |  |  |  |  |  |

Table A2: Comparison of low extremity joint angle of the participant between conditions

| Walking Condition | Without IDEO | | With IDEO | |
| --- | --- | --- | --- | --- |
| Leg side | Left | Right | Left | Right |
| Ankle flexion Max (deg) | 20(1.7) | 11(1.5) | 7(0.8) | 10(1.9) |
| Ankle flexion Min (deg) | 0(3.3) | (-)23(2.1) | (-)2(0.4) | (-)24(3.1) |
| Ankle flexion ROM (deg) | 20(2.8) | 34(3.1) | 9(0.8) | 34(2.7) |
| Hip flexion Max (deg) | 44(2.6) | 41(1.0) | 43(1.7) | 41(1.4) |
| Hip flexion Min (deg) | 7(1.1) | 3(0.7) | 2(1.4) | 6(1.0) |
| Hip flexion ROM (deg) | 37(2.8) | 38(1.2) | 41(2.1) | 35(1.6) |
| Knee flexion Max (deg) | 70(1.2) | 61(1.1) | 60(2.6) | 70(1.3) |
| Knee flexion Min (deg) | 4(1.4) | (-)2(0.6) | (-)6(1.3) | 1(1.5) |
| Knee flexion ROM (deg) | 65(1.5) | 63(1.3) | 66(2.6) | 68(2.4) |

Note: data are presented as mean (standard deviation)
